# Substance Related and Addictive Disorders in Sports with a Focus on the Football Sector: a Narrative Review

**DOI:** 10.1192/j.eurpsy.2022.2135

**Published:** 2022-09-01

**Authors:** S. Toparlak, D. Gurrea Salas

**Affiliations:** 1 Oxford Health NHS Foundation Trust, Psychiatry, Oxford, United Kingdom; 2 Psychiatric Services Aargau AG (PDAG), Psychiatry, aarau, Switzerland

**Keywords:** sports medicine, Addiction, addictive disorders, football

## Abstract

**Introduction:**

Football is the world’s most-watched and played sport. Even though sports psychiatry is steadily gaining importance, the stigma on mental illness in sports, especially football, and the limited number of articles on this topic means there is a pressing need for more study in this area. This narrative review begins to fill this gap. This review summarises the work on addictive disorders in sports, with a close focus on football, as well as mentioning some initiatives that are advancing our understanding of how mental illnesses in sports can be addressed.

**Objectives:**

This view also contributes to understanding the reasons behind mental illness and sports, and raises awareness.

**Methods:**

This review was conducted by searching for the keywords ‘addiction’ and ‘football’ on three different database search engines, namely, PubMed, Cochrane Library, and Medline. We found 26 articles based on this literature search with these keywords from 2005 to 2020. After data extraction, we cited 10 of them considering the specificity of addiction disorders in the football industry. 16 additional articles found by backwards citation chaining are also included in this review.

**Results:**

The articles reviewed here investigate addictive disorders within the football sector by looking at the incidence of particular addictive disorders, their underlying reasons and their consequences. This piece concludes by showing the need for more research and new initiatives regarding addictive disorders within the target group of footballers.

**Conclusions:**

A holistic, multidisciplinary and biopsychosocial approach is essential to provide long term solutions considering different factors contributing to addictive disorders in the football sector.

**Disclosure:**

No significant relationships.

